# Online Measurement of Exhaled NO Concentration and Its Production Sites by Fast Non-equilibrium Dilution Ion Mobility Spectrometry

**DOI:** 10.1038/srep23095

**Published:** 2016-03-15

**Authors:** Liying Peng, Dandan Jiang, Zhenxin Wang, Jiwei Liu, Haiyang Li

**Affiliations:** 1 Key Laboratory of Separation Science for Analytical Chemistry, Dalian Institute of Chemical Physics, Chinese Academy of Sciences, Dalian, 116023, People’s Republic of China; 2 University of Chinese Academy of Sciences, Beijing, 100049, People’s Republic of China; 3 Institute of Chemistry for Functionalized Materials, Faculty of Chemistry and Chemical Engineering, Liaoning Normal University, Dalian 116029, China

## Abstract

Exhaled nitric oxide (NO) is one of the most promising breath markers for respiratory diseases. Its profile for exhalation and the respiratory NO production sites can provide useful information for medical disease diagnosis and therapeutic procedures. However, the high-level moisture in exhaled gas always leads to the poor selectivity and sensitivity for ion spectrometric techniques. Herein, a method based on fast non-equilibrium dilution ion mobility spectrometry (NED-IMS) was firstly proposed to directly monitor the exhaled NO profile on line. The moisture interference was eliminated by turbulently diluting the original moisture to 21% of the original with the drift gas and dilution gas. Weak enhancement was observed for humid NO response and its limit of detection at 100% relative humidity was down to 0.58 ppb. The NO concentrations at multiple exhalation flow rates were measured, while its respiratory production sites were determined by using two-compartment model (2CM) and Högman and Meriläinen algorithm (HMA). Last but not the least, the NO production sites were analyzed hourly to tentatively investigate the daily physiological process of NO. The results demonstrated the capacity of NED-IMS in the real-time analysis of exhaled NO and its production sites for clinical diagnosis and assessment.

Exhaled nitric oxide (NO) is derived from an alveolar and an airway source, that is, NO with low solubility in aqueous solutions escapes into the airways and is transported in the exhaled gas from the alveolar region; on the way out, it collects additional NO diffusing from the airway walls^1^,^2^,^3^. The fraction of NO in exhaled gas (F_E_NO) is not only highly correlated with eosinophilic airway inflammation but also positively predicts the steroid treatment^4^,^5^, which has been proved to be useful in diagnostic as well as in therapeutic procedures. However, using only one flow rate is not sufficient to reveal the origin of NO and its release mechanism to the exhaled gas. According to the previous researches, the discovery of the flow dependence of exhaled NO sheds light on modeling the NO production sites in respiratory system and its release mechanism to the exhaled gas^6^,^7^,^8^,^9^. The two-compartment model (2CM) based on Fick’s first law of diffusion is most commonly used while many algorithms have been developed to calculate it^10^,^11^. Among them, linear and nonlinear algorithms were frequently described in the literatures^12^. For example, Tsoukias and George proposed a linear technique (T&G) to calculate the 2CM^13^, while Högman and Meriläinen found a nonlinear algorithm (HMA), by which all of the flow-independent NO exchange parameters can be determined^14^. Nevertheless, determining the NO production sites with 2CM and its analytical algorithms request exhaled NO concentrations at different exhalation flow rates. Thus, it is significant to find a way to detect exhaled NO at different exhalation flow rates.

Many techniques including chemiluminescence analyzers, electrochemical sensors and laser-based techniques have been developed to detect exhaled NO^15^,^16^,^17^. Chemiluminescence analyzers are considered as the standard technique and have been widely adopted for online analysis due to its fast response time (0.5–0.7 s). Nevertheless, the complete reduction of NO_x_ to NO and its oxidation with ozone increases the operational complexity. Other shortcomings of chemiluminescence also include bulky size, high running costs and requirement of technical expertise for calibration, which also limit their routine use^17^,^18^. Even though electrochemical sensors are in favor of development of portable or even hand-held devices such as the amperometric sensors developed by Aerocrine (NIOX-MINO), the device sensor needs to be replaced after 100–300 actuations resulting in the performances change over time^19^. More importantly, it is not suitable for multiple flow analysis, and the relatively long response time even makes them to only obtain the average F_E_NO value for an exhalation^20^,^21^. Laser-based methods such as quantum cascade laser (QCL) technologies etc., were also developed for the detection of NO at low ppb level^22^. In spite of high selectivity to the target compounds and fast response time, they are suffering from the expensive cost, spectral degradation and reliability problems of laser source^23^,^24^,^25^. Recently, efforts are also being devoted to developing other feasible techniques to measure exhaled NO^26^. For example, Pan *et al*. proposed a method based on extractive electrospray ionization mass spectrometry (EESI-MS) for quantitative detection of exhaled NO at ppb level^27^. The F_E_NO could be derived from the EESI-MS response of the product of selective reaction between 2-phenyl-4, 4, 5, 5-tetramethylimidazoline-1-oxyl-3-oxide (PTIO) reagent and NO molecules. However, the average time of 150 s for each sample collection and the usage of solvents hindered its application for online measurement. Thus, other techniques with features of easy portability, simple operation, high sensitivity and rapid response are in demand for online monitoring of exhaled NO.

Ion mobility spectrometry (IMS) has been utilized in breath analysis, where speed, cost and specificity of IMS are viewed as strong advantages, particularly in process monitoring^26^,^28^. When it is applied to breath analysis, however, the high moisture in exhaled gas would significantly complicate the ion spectra and result in the poor selectivity and sensitivity^29^. To date, IMS coupled with a multi-capillary column (MCC) or gas chromatography (GC) is an effective way to keep the moisture away, but the analysis time of 600 s makes it difficult for the online monitoring^26^,^30^. More recently, we developed a method based on the dopant titrating (DT) IMS to measure exhaled NO^31^. The interference of moisture was eliminated by introducing a dopant, p-benzoquinone (PBQ), into IMS to titrate the interfering ion peaks for impurities. The time of 4 s for sample collection, however, limited it to measure the average F_E_NO value for an exhalation, and hampered the monitoring of F_E_NO profile versus time, where a stable NO plateau can be exhibited. According to the recommendations of European Respiratory Society and American Thoracic Society (ATS/ERS), the exhaled NO content should be derived from the plateau concentration evaluated over a 3-second window of the exhalation profile^32^. Hence, a faster method based on IMS should be developed for online measurement of NO within single-breath profile.

Herein, a novel method based on fast non-equilibrium dilution ion mobility spectrometry (NED-IMS) was proposed to capture the exhaled NO profile in real time. The moisture interference on the sensitivity was investigated in detail while exhaled NO at different exhalation flow rates was measured. Based on 2CM and HMA, the NO production sites in respiratory system were determined, while its change during a day was studied as well.

## Methods

### Apparatus

**Figure 1:**
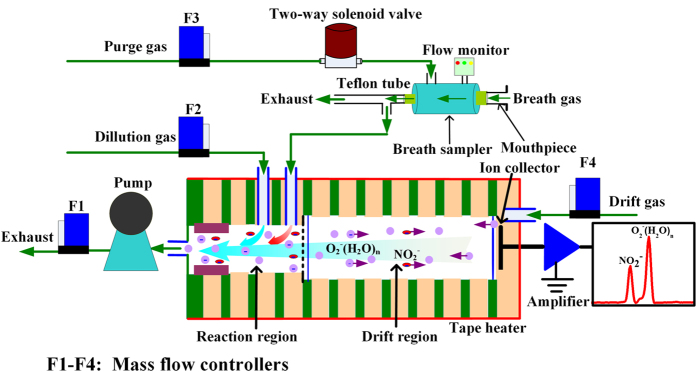
Schematic diagram of fast non-equilibrium dilution ion mobility spectrometer.

A schematic diagram of the fast non-equilibrium dilution ion mobility spectrometer with automatic sampling system was demonstrated in Fig. 1. Different from the IMS reported previously^31^, an inlet in the reaction region was designed for sampling while an extra inlet was punched to introduce a dilution gas. The inlet located in front of the ionization source was used as the outlet, before which a gas pump was adopted for exhausting air. The flow rate of pumping, dilution gas and drift gas controlled at 1.0 L min^−1^, 200 mL min^−1^ and 600 mL min^−1^ via mass flow controllers (Beijing Sevenstar Electronics Co., Ltd), respectively. Thus, the sampling flow rate could be kept at 200 mL min^−1^. Dry air purified by silica gel, activated carbon and 13× molecular sieve traps was divided into drift gas and dilution gas. Another purified gas was chosen to purge the breath sampler and controlled via a two-way solenoid valve that was shut down during the breathing (sampling) and was opened as the breath is over. In addition, the drift tube temperature was controlled at 90 °C by a tape heater. The measurement time of IMS was 75 ms (the average of five times acquisition), while the fast data acquisition software with a repetition frequency of ~13 Hz was developed.

Due to the flow dependence of exhaled NO^32^, a homemade breath sampler was developed for controlling the exhalation flow rates. The main body of breath sampler was a tube (φ8 mm) made of PEEK (polyether-ether-ketone), which was assembled from a flow monitor, a mouthpiece, and a Teflon tube (Φ4 mm) for expiratory resistance. The Teflon tube length of 100 cm and 1 cm was designed to obtain different expiratory resistances for the low flow rates and high flow rates, respectively. The relations between the values of flow monitor and exhalation flow rates were calibrated and a wide-range flow rates could be obtained. When sampling process started, volunteers took a deep breath of purified gas firstly, avoiding the disturbance of NOx in air, and then performed a single breath through the mouthpiece. Feedbacks were given to volunteers via three LEDs to help them to maintain mouth pressure around the set values, which were corresponding to different exhalation flow rates.

### Breath samples

All experimental protocols were approved by the Ethics Committee of the Dalian Institute of Chemical Physics, Chinese Academy of Sciences and the methods were carried out according to the approved guidelines. All the subjects (volunteers) had been informed the content of this experiment before their breath tests, and informed consents were obtained from the involved subjects. Thirteen volunteers including five women and eight men (including one regular smoker) maintained their normal daily lifestyle during the test. Among them, three volunteers took part in the hourly measurement of exhaled NO in three days.

### Materials

Standard gas of 10000 ppm NO balanced with N_2_ was purchased from Dalian Great Special Gas Co., Ltd (Dalian, China). NO samples with different concentrations were prepared by successively diluting 10000 ppm NO standard gas with purified dry air and humid air. Humid air with well-defined relative humidity (RH) was generated by mixing the dry air and the saturated air (100% RH), which was obtained through bubbling, as reported by Vautz *et al*.^33^. The level of moisture was monitored by a dew-point hygrometer (CS Messtechnik GmbH).

### Two-compartment model (2CM)

When the F_E_NO at different flow rates was detected in virtue of the breath sampler, the NO production sites in the respiratory system can be calculated with the two-compartment model (2CM). The 2CM based on Fick’s first law of diffusion consists of one rigid compartment and one expansile compartment, which represent the conducting airways and gas-exchange alveolar region of lung, respectively^34^. The NO transfer from the alveoli or airway tissue to airway lumen is controlled by the Fick’s first law of diffusion^35^. The NO production sites in the respiratory system are defined with four different parameters including the fraction of NO in the gas-phase alveolar region (C_A_NO in ppb) produced by the expansile compartment of lung, the airway tissue concentration of NO (wall concentration, C_aw_NO in ppb) released by the rigid conducting airway system, the transfer factor indicating the total airway compartment diffusion capacity (D_aw_NO in pL s^−1^ ppb^−1^ or mL s^−1^) and the flux of NO from the airway wall to the lumen (J_aw_NO in pL s^−1^)^36^. The correlation between the parameters and exhaled NO is presented in equation (1), where V_E_ is the exhalation flow rate (mL s^−1^). The parameter of J_aw_NO can be characterized by equation (2), whose value reaches to the maximum as C_A_NO is zero.


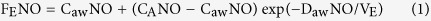



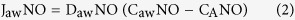


### Högman and Meriläinen algorithm (HMA)

Högman and Meriläinen algorithm (HMA) is frequently used to extract the parameters of J_aw_NO, C_A_NO, D_aw_NO and C_aw_NO^10^,^12^,^14^. In this algorithm, F_E_NO values at three flow rates, i.e., low (≤20 mL s^−1^), medium (100 mL s^−1^) and high (300–500 mL s^−1^) V_E_, are measured. The two parameters of C_A_NO and J_aw_NO can be estimated from the linear plot of V_NO_ versus medium and high V_E_. In theory, when V_E_ > ~5 × D_aw_NO mL s^−1^ or 50 mL s^−1^, equation (1) is transformed to equation (3) by Taylor’s approximation, and V_NO_ can be obtained from equation (4), the slope and the intercept from the plot of V_NO_ versus V_E_ yield C_A_NO and J_aw_NO, respectively. Parameters of D_aw_NO and C_aw_NO are then calculated using F_E_NO values at three flows by solving equation (1) and (2) through an iterative algorithm.


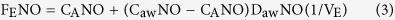



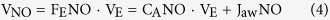


## Results and Discussion

### Real-time monitoring of exhaled NO profile

**Figure 2:**
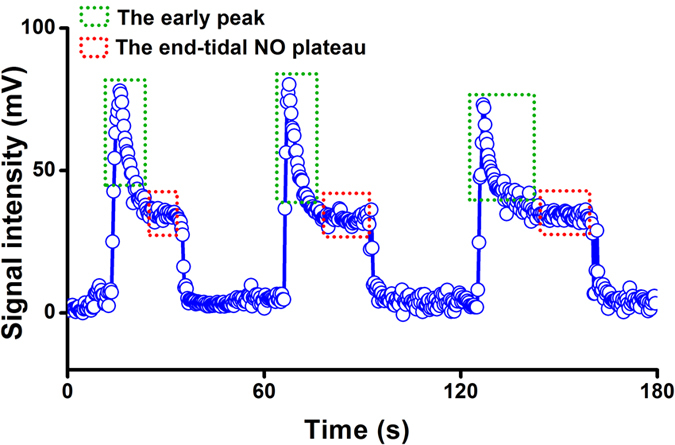
Exhaled NO profiles for three separate exhalations of the same subject at the flow rate of 50 mL s^−1^.

When the exhalation flow rate was stabilized at 50 mL s^−1^, the ion mobility spectrum of breath gas was measured in Supplementary Fig. S1. From the spectrum, we learned that the NO ion peak was appeared at 2.60 cm^2^ V^−1^ s^−1^ while other substances in the breath gas had little effect on the identification. The ion mobility detected here was different from that obtained by the dopant titrating method (*K*_0_ = 2.43 cm^2^ V^−1^ s^−1^) due to the higher tube temperature of 90 °C. With the IMS response time of 75 ms, the profiles for three separate exhalations of the same subject could be monitored in Fig. 2. The NO profile for a single-breath in Fig. 2 consisted of an early NO peak then followed by an NO plateau. On account of the purified inhaled air (without exogenous NO) and inhalation from the mouth, the early NO peak might be derived from the nasal cavity due to the velum initial opening as the exhalation starts^32^. Hence, the early peaks should be ignored, while only NO plateaus were used to calculate the exhaled NO concentration. What is more, from the profiles, we can know that the signal intensity for three exhalations was 34.58 mV, 33.77 mV and 34.97 mV, respectively, with the average value of 34.44 mV and the relative standard deviation (RSD) of 1.78%, which demonstrated the good reproducibility for the detection.

### Reducing the interference of moisture by non-equilibrium dilution

**Figure 3:**
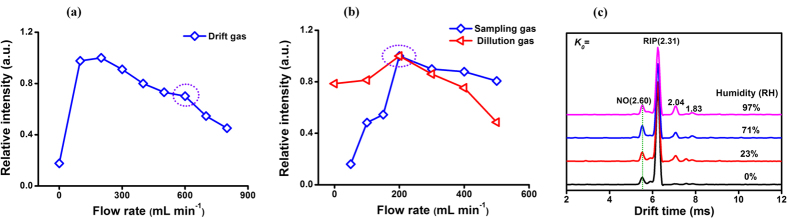
(**a**) The curve for signal intensity of 50 ppb NO in 95% RH humid air versus drift gas flow rate; (**b**) the curves for signal intensity of 50 ppb NO in 95% RH humid gas versus sampling gas and dilution gas flow rate; (**c**) ion mobility spectra of 25 ppb NO in different humidity gas.

In order to investigate the effect of drift gas on the experimental signal, the signal intensities of 50 ppb 95% RH humid NO at different flow rates of drift gas were detected in Fig. 3(a). Even though the response for NO reached to the maximum at drift gas of 200 mL min^−1^, many interfering peaks related to moisture in spectra would hamper its qualitative identification and quantification (Supplementary Fig. S2). In view of these two aspects, drift gas of 600 mL min^−1^ was eventually chosen for the next study. Similarly, both dilution gas and sampling gas were optimized in Fig. 3(b). Their curves all exhibited the maximum intensity for 50 ppb NO at the flow rate of 200 mL min^−1^. Thus, 200 mL min^−1^ was chosen for both sampling gas and dilution gas. At the above optimizing flow rates, different humidity samples were detected and the ion mobility spectra for 25 ppb NO were demonstrated in Fig. 3(c). In the spectra, the drift time of NO appeared at 2.60 cm^2^ V^−1^ s^−1^, while the main interfering peaks from impurities among moisture were at *K*_*0*_ of 2.04 cm^2^ V^−1^ s^−1^ and 1.83 cm^2^ V^−1^ s^−1^. The interfering peaks were far away from the product ion peak of NO and did not affect the identification. Meanwhile, the signal intensities variation for 25 ppb and 50 ppb NO in Supplementary Fig. S3 appeared a weak enhancement for humid NO response indicating that the moisture at a certain extend promotes the ionization to improve the sensitivity in the NED-IMS.

When the moistures in sample gas and exhaust gas were monitored, the curve of exhaust gas versus sample gas was plotted in Supplementary Fig. S4. From the curve, we could learn that the moisture of samples in the reaction region was lowered to 21% of the original. The dilution ratio of moisture in the samples (79%) was almost the same as the volume ratio per minute of drift gas and dilution gas in the reaction region (80%). In addition, it is noteworthy that the gas flow in the reaction region, especially around the inlet or outlet, is in a turbulence which is a non-equilibrium state in space^37^. As the gas flow moving to the outlet of IMS, the humidity in the reaction region is gradually reduced by the turbulent mixing of dilution gas and drift gas, greatly weakening the interference of moisture on the ionization efficiency of NO. Also, the drift gas (0% RH) in the drift region ensured the ions migrating to the ion collector without severe cluster reaction. Consequently, the interference of moisture in humid gas even exhaled gas would be eliminated by the turbulent dilution of drift gas and dilution gas.

### Quantitation of NED-IMS

Under the optimal conditions, the quantitative analysis for the humid samples was performed and the results in the Supplementary Table S1 demonstrated that the linear range expanded from 4–140 ppb to 5–180 ppb as the moisture in samples increasing from 0% RH to 100% RH with the RSD (n = 9) of less than 5.78%. Unlike the decreasing tendency at elevated moisture obtained by DT-IMS^31^, the calculated limits of detection (LODs, S/N = 3) reached the lowest value of 0.35 ppb at 70% RH, while 0.58 ppb were obtained for the 100% RH sample, as seen in Table 1. The sensitivity of 100% RH sample detected by NED-IMS was enhanced by a factor of 2.4 than that of TD-IMS. Thus, the sensitivity of this method was further improved for the diagnosis requirement for F_E_NO in health individuals (8 ppb)^22^, which is significantly lower than that of patients with respiratory diseases (>25 ppb in adults, 20 ppb in children)^38^.

### The exhaled NO at different exhalation flow rates

**Figure 4:**
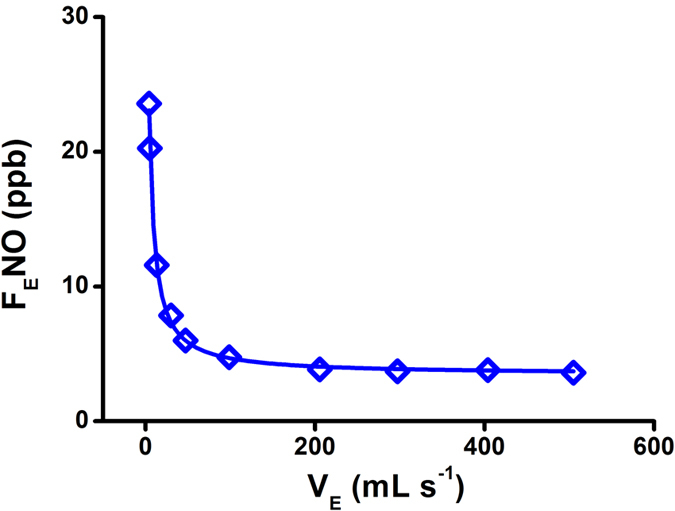
The profile for the F_E_NO at different exhalation flow rates.

In order to obtain the breath gas at different exhalation flows, two different expiratory resistances for breath sampler were fixed by changing the length of Teflon tube (Φ4 mm). The length of 100 cm and 1 cm was chosen for the low flow rates and high flow rates, respectively. The low flow rates could be obtained through the calibration curve of flow rates versus the feedback of pressure in Supplementary Fig. S5(a), while the high flow rates could be obtained from that in Supplementary Fig. S5(b). Thus, the exhaled NO at different exhalation flow rates were detected, while its profile for a volunteer was demonstrated in Fig. 4. The F_E_NO calculated with the quantitative equation at 100% RH exponentially damped from 24 ppb to 3.5 ppb as the exhalation flow rates increasing from 4 mL s^−1^ to 505 mL s^−1^, which was consistent with what reported previously^39^. The results demonstrated the capability of current NED-IMS for analysis F_E_NO at a wide-range exhalation flow rates.

### The exhaled NO production sites analyzed with HMA model

According to the nonlinear HMA model, the F_E_NO values at three exhalations flow rates of 5 mL s^−1^, 100 mL s^−1^ and 500 mL s^−1^ were used to analysis of the NO production sites in respiratory system. The production parameters including J_aw_NO (pL s^−1^), C_aw_NO (ppb), D_aw_NO (pL s^−1^ ppb^−1^) and C_A_NO (ppb) for thirteen healthy volunteers were obtained, as listed in Table 2. From Table 2, we can learn that the four parameters were different from each other: the J_aw_NO values ranged from 95.5 pL s^−1^ to 831.7 pL s^−1^; D_aw_NO values ranged from 7.2 pL s^−1^ ppb^−1^ to 37.2 pL s^−1^ ppb^−1^; the C_aw_NO values ranged from 6.0 ppb to 32.0 ppb; and the C_A_NO values ranged from 1.5 ppb to 3.0 ppb. Based on our results, the J_aw_NO and D_aw_NO levels of males were generally higher than those of females, while C_aw_NO and C_A_NO levels for males and females had no significant difference. Compared with the previous reports, the levels of four parameters were generally in line with the results of low group A and the clinical setting obtained by CLD 88sp NO analyzer system (chemiluminescence, ECO Medics AG, Duernten, Switzerland)^12^. While the range of values of J_aw_NO, D_aw_NO and C_A_NO obtained in this study are intersections with the parameters obtained by NOA 280 analyzer (chemiluminescence, Sievers, USA)^9^,^40^, which demonstrated the reliability of NED-IMS.

### The changes of exhaled NO production during one day

**Figure 5:**
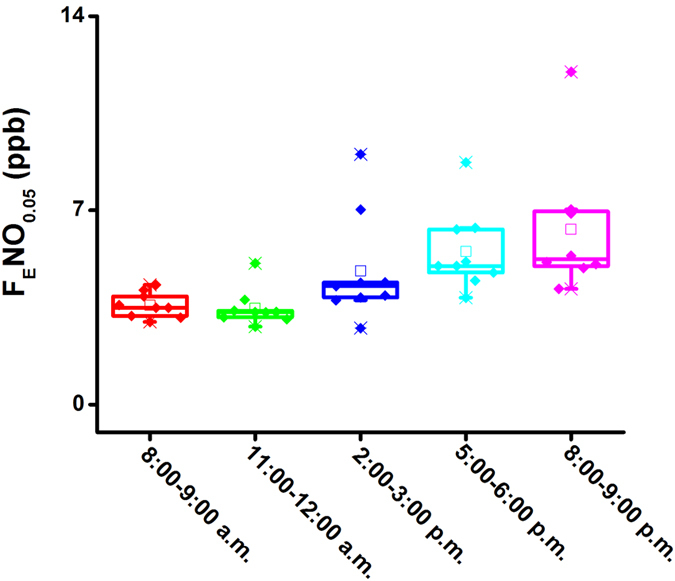
Box-and-whisker plots for hourly measurement of exhaled NO at 50 mL s^−1^ of three volunteers in three days (from 8:00 a.m. to 9:00 p.m.). Box-and-whisker plots: the bottom and top of the box present the first and third quartile, respectively; the band inside the box is always the second quartile (the median); lines extending vertically from the boxes (whiskers) stand for the upper and lower extreme (the highest and lowest number in a set of data) and also indicate the variability outside the upper and lower quartiles.

**Figure 6:**
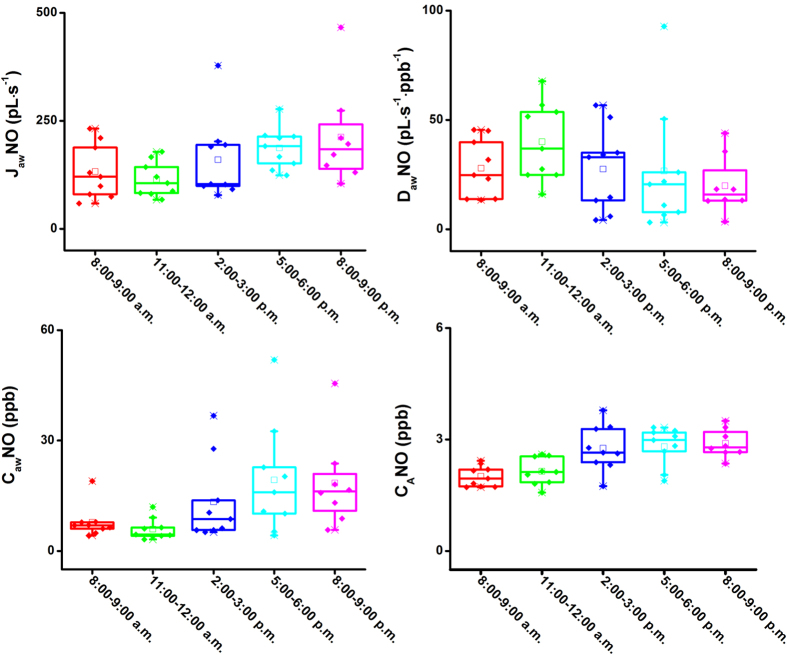
Box-and-whisker plots for four parameters obtained by HMA model within three days (from 8:00 a.m. to 9:00 p.m.).

The exhaled NO at 50 mL s^−1^ for three volunteers were detected hourly in three days (twelve hours, from 8:30 a.m. to 9:00 p.m.), and their box-and-whisker plots are depicted in Fig. 5. From the plots, we found that the average F_E_NO levels were lowest in the morning, approximately 3.5 ppb, and then progressively increased to 6.32 ppb at the period of 8:00–9:00 p.m. This tendency is generally in line with that obtained by DT-IMS^31^ and is also consistent with the consequence (exhaled NO values in afternoon were higher than morning values) reported in previous literatures^41^,^42^. It might be resulted from the impact of human metabolic state, physiologic parameters and other factors such as food and beverages. In order to investigate further the physiological process of NO in the respiratory system, the exhaled NO production sites within three days were detected, and the box-and-whisker plots for the four parameters were depicted in Fig. 6. The parameters of J_aw_NO, C_aw_NO and C_A_NO increased from 132 pL s^−1^ to 217 pL s^−1^, 8.4 ppb to 18.5 ppb and 2.0 ppb to 2.9 ppb, respectively, while D_aw_NO increased firstly to the maximum of 40.2 pL s^−1^ ppb^−1^ (11:00–12:00 a.m.), then decreased to 19.5 pL s^−1^ ppb^−1^ (8:00–9:00 p.m.). Except for D_aw_NO, the change tendencies for J_aw_NO, C_aw_NO and C_A_NO coincided with that of F_E_NO at 50 mL s^−1^. Thus, the factors of human metabolic state, physiologic parameters and other factors such as food and beverages were essentially impact on the pulmonary alveoli and airway condition, and then finally reflected on the F_E_NO level.

## Conclusion

In summary, exhaled NO during an exhalation was monitored in real time by a non-equilibrium dilution ion mobility spectrometer with response time of 75 ms while its profiles at different flow rates were obtained. The moisture influence on the detection was greatly weakened through the non-equilibrium dilution of drift gas and dilution gas, and the LOD for NO in 100% RH purified air was down to 0.58 ppb. In combination with the 2CM as well as HMA, the NO production sites in respiratory system was obtained and successfully applied to explain the variation of exhaled NO during one day. Consequently, the currently established method based on IMS was demonstrated to be a powerful technique for the real-time measurement of exhaled NO and the analysis of NO production sites for clinical diagnosis and assessment.

## Additional Information

**How to cite this article**: Peng, L. *et al*. Online Measurement of Exhaled NO Concentration and Its Production Sites by Fast Non-equilibrium Dilution Ion Mobility Spectrometry. *Sci. Rep*. **6**, 23095; doi: 10.1038/srep23095 (2016).

## Supplementary Material

Supplementary Information

## Figures and Tables

**Table 1:** Comparison of limits of detection (LODs) obtained by NED-IMS and DT-IMS.

Humidity (%, RH)	LODs (ppb, S/N = 3)	
	NED-IMS	DT-IMS^31^
0	0.42	0.51
30	0.59	0.80
70	0.35	1.00
100	0.58	1.40

**Table 2:** The four parameters for thirteen volunteers obtained by the HMA model.

Time	Volunteers	J_aw_NO pL s^−1^	D_aw_NO pL s^−1^ ppb^−1[a]^	C_aw_NO ppb	C_A_NO ppb
Morning	Female (25)	95.5	18.8	7.3	2.3
	Female (27)	126.1	7.2	20.1	2.6
	Male (25)	159.2	37.2	6.0	1.7
	Male (25)	142.1	19.9	8.9	1.7
	Male (35, smoking)	151.6	18.3	10.7	2.4
Afternoon	Male (31)	200.1	17.9	13.2	2.0
	Male (30)	203.2	23.1	10.3	1.5
	Male (26)	288.7	26.8	13.3	2.5
	Male (26)	680.9	25.8	28.7	2.4
	Male (28)	206.1	11.0	21.2	2.5
	Female (26)	831.7	28.4	32.0	2.7
	Female (26)	99.2	8.9	13.6	2.4
	Female (25)	191.3	17.2	14.1	3.0

^[a]^pL s^−1^ ppb^−1^ is the same as mL s^−1^.
