# Supplementary material for: Online Measurement of Exhaled NO Concentration and Its Production Sites by Fast Non-equilibrium Dilution Ion Mobility Spectrometry
Source: Sci Rep. 2016 Mar 15;6:23095. doi: 10.1038/srep23095 (PMC4791560; doi:10.1038/srep23095)
Supplement: Supplementary Information [file srep23095-s1.doc]

**Supplementary Information**

Online Measurement of Exhaled NO Concentration and Its Production Sites by Fast Non-equilibrium Dilution Ion Mobility Spectrometry

Liying Peng,^†,‡^ Dandan Jiang, ^†,‡^ Zhenxin Wang,^†^ Jiwei Liu^§^ and Haiyang Li*^,†^

^†^Key Laboratory of Separation Science for Analytical Chemistry, Dalian Institute of Chemical Physics, Chinese Academy of Sciences, Dalian, 116023, People’s Republic of China

^‡^University of Chinese Academy of Sciences, Beijing, 100049, People’s Republic of China

^§^Institute of Chemistry for Functionalized Materials, Faculty of Chemistry and Chemical Engineering, Liaoning Normal University, Dalian 116029, China

*E-mail: hli@dicp.ac.cn. Fax: +86-411-84379517.

**SI 1. Detection of NO in exhaled gas**

The ion mobility spectrum of breath gas was demonstrated in Fig. S1 as the exhalation flow rate was stabilized at 50 mL s^-1^. In the spectrum, the NO ion peak was appeared at 2.60 cm^2^ V^−1^ s^−1^ while other substances in breath gas did not affect the identification. The ion mobility detected here was different from that obtained by the dopant titrating method due to the higher tube temperature.


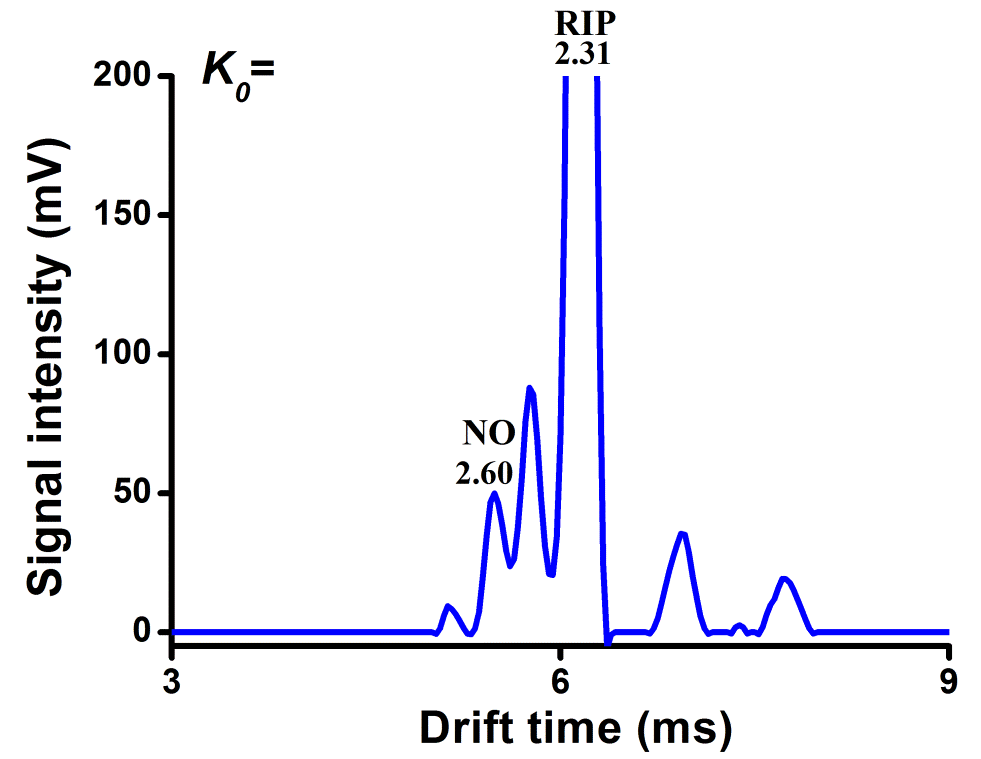


**Supplementary Figure S1.** Ion mobility spectrum of exhaled gas of volunteer.

**SI 2. Influence of drift gas flow rate**

The ion mobility spectra of 50 ppb NO in 95% RH humid gas at different drift gas flow rates were demonstrated in Fig. S2. As the drift gas flow rate increasing, ion peaks of impurities related to moisture weakened, simplifying the spectra and benefiting for the identification and quantification of humid NO.


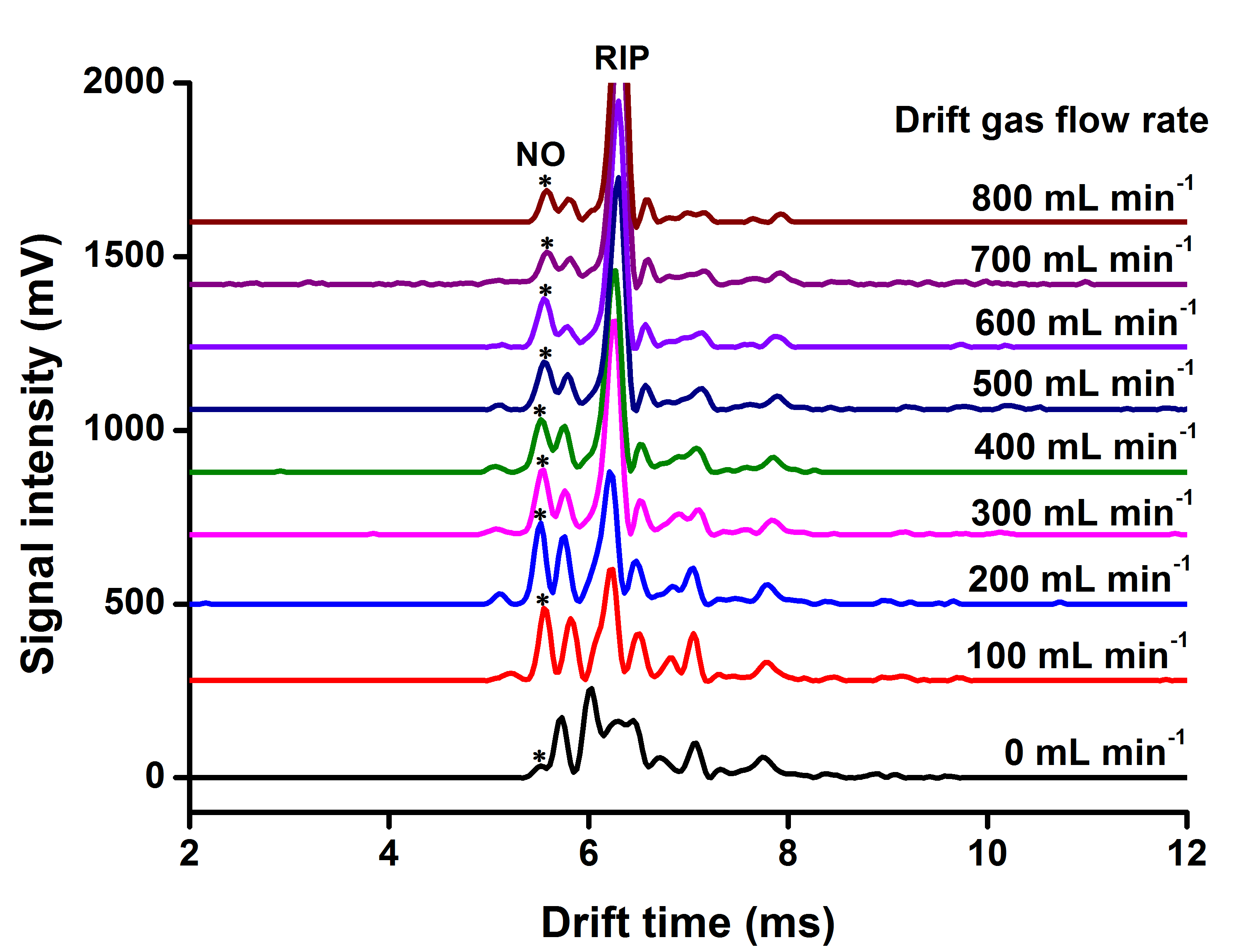


**Supplementary Figure S2.** Ion mobility spectra of 50 ppb NO in 95% RH humid gas at different drift gas flow rates.

**SI 3. Influence of dilution gas and sampling gas flow rate**

The signal intensity variation curves for 25 ppb and 50 ppb NO at different humidity in Fig. S3 demonstrated that their intensities were enhanced weakly as the humidity increasing to 40% RH, and then slightly changed as it continuously increasing, while the reactant ion peak (RIP) intensity decreased from 880 mV to 610 mV (from 0% RH to 97% RH). The signal enhancement indicated that the moisture at a certain extend promotes the ionization to improve the sensitivity in the NED-IMS.


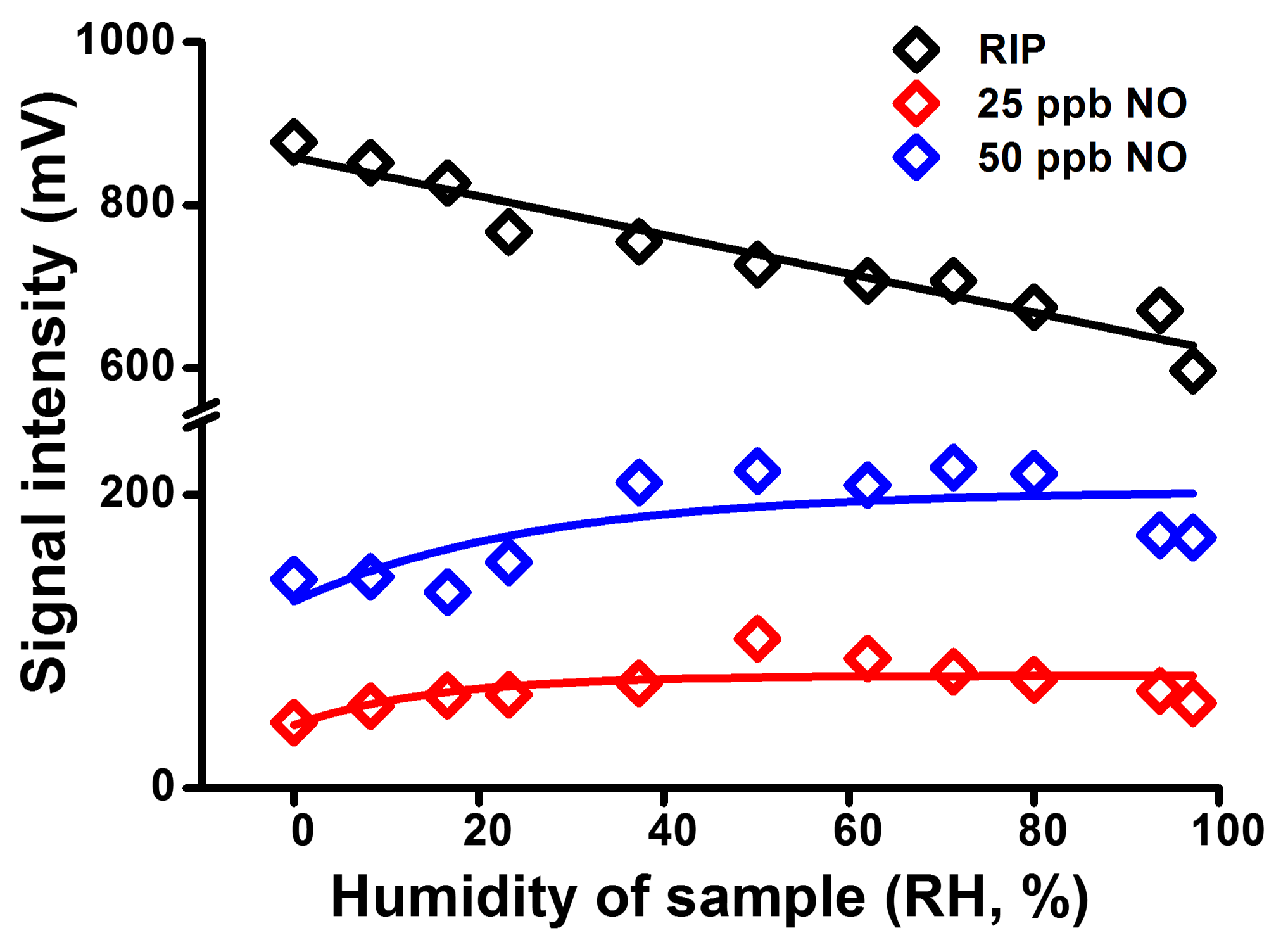


**Supplementary Figure S3.** The signal intensity variations for 25 ppb and 50 ppb NO at different humidity of samples.

**SI 4. The dilution ratio by drift gas and purified gas**

When the moistures in sample gas and exhaust gas were monitored, the curve of exhaust gas versus sample gas was plotted in Fig. S4. The moisture of samples in the reaction region was lowered to 21% of the original. The dilution ratio of moisture in the samples (79%) was almost the same as the volume ratio per minute of drift gas and dilution gas in the reaction region (80%).


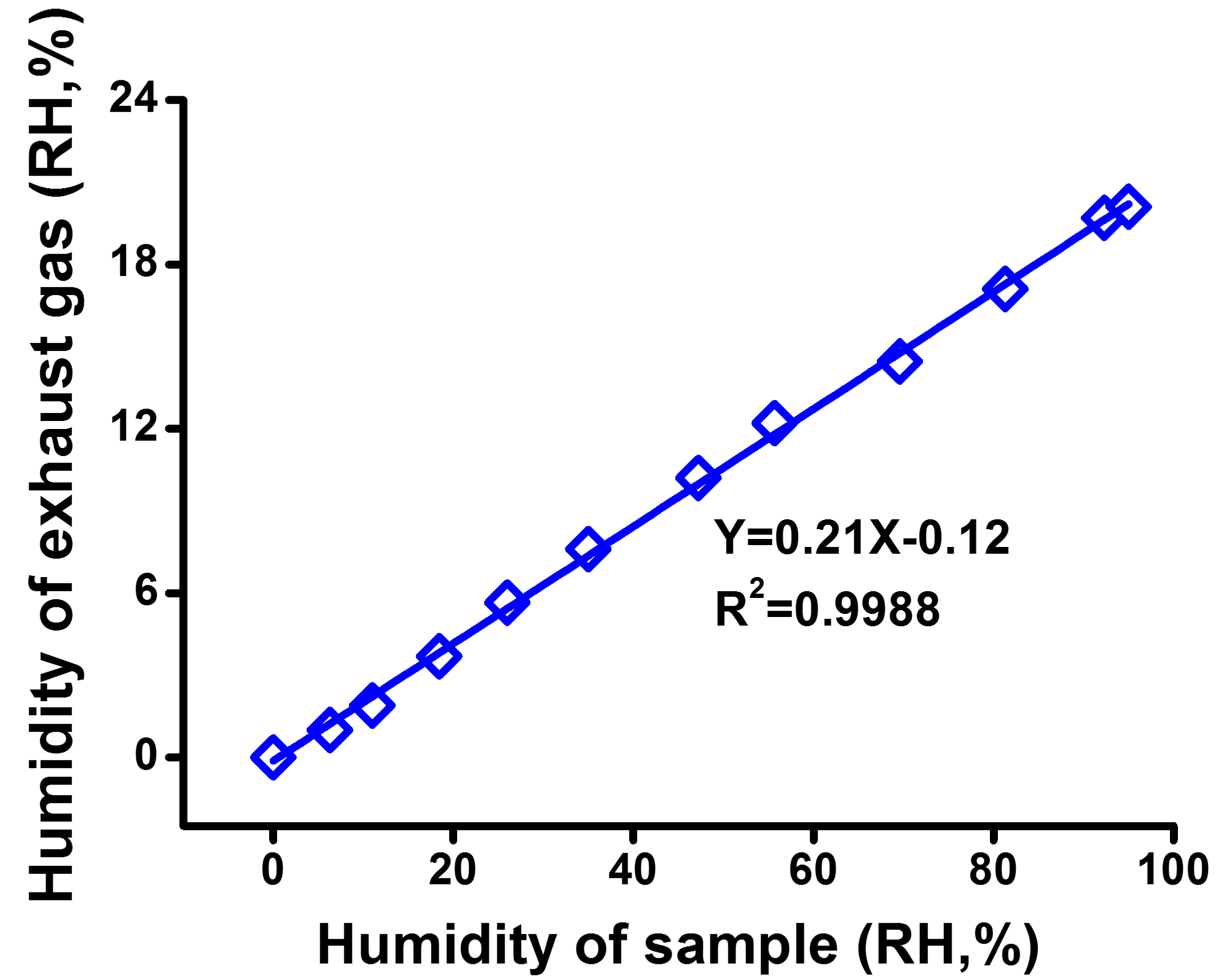


**Supplementary Figure S4.** The curve for the moisture in exhaust gas and humid sample gas.

**SI 5. Calibration of breath sampler**

The low flow rates could be obtained through the calibration curve of flow rates versus the feedback of pressure in Fig. S5(a), while the high flow rates could be obtained from that in Fig. S5(b).


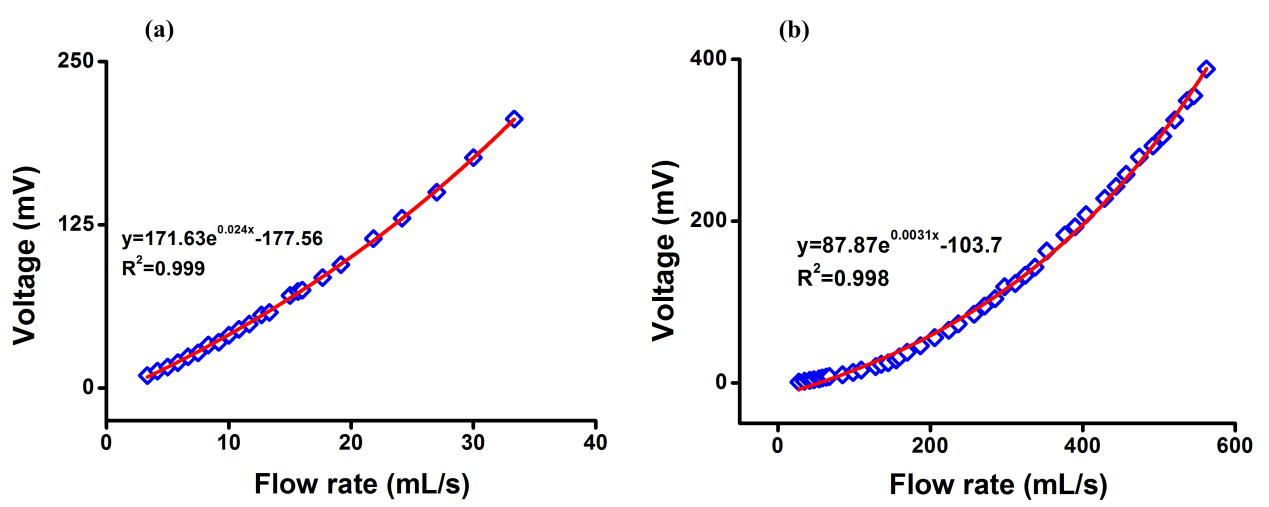


**Supplementary Figure S5.** The calibration curves for the (a) low flow rates and (b) high flow rates.

**SI 6. The quantitation analysis**

**Supplementary Table S1.** The quantitative results in different humidity.

| Humidity (%, RH) | 0 | 30 | 70 | | 100 | |
| --- | --- | --- | --- | --- | --- | --- |
| Linear Function | y=2.92x+0.23 | y=2.76x+2.06 | y=3.44x+7.23 | | y=2.83x+0.87 | |
| R^2^ | 0.991 | 0.985 | 0.990 | | 0.989 | |
| Liner Range (ppbv) | 4-140 | 5-140 | 5-140 | | 5-180 | |
| LOD (ppbv, S/N=3) | 0.42 | 0.59 | 0.35 | | 0.58 | |
| RSD (%) | 2.27 | 5.78 | 5.02 | 5.05 | |  |
